# PGK1 contributes to tumorigenesis and sorafenib resistance of renal clear cell carcinoma via activating CXCR4/ERK signaling pathway and accelerating glycolysis

**DOI:** 10.1038/s41419-022-04576-4

**Published:** 2022-02-04

**Authors:** Yu He, Xixi Wang, Weiliang Lu, Dan Zhang, Lan Huang, Yang Luo, Li Xiong, Haocheng Li, Peng Zhang, Qiu Li, Shufang Liang

**Affiliations:** 1 State Key Laboratory of Biotherapy and Cancer Center, West China Hospital, Sichuan University, Chengdu, 610041 P. R. China; 2 Department of Pharmacy, The First Affiliated Hospital of Chongqing Medical University, Chongqing, 400016 China; 3 Department of Mathematics and Statistics, University of Calgary, Calgary, AB T2N 1N4 Canada; 4 Department of Urinary Surgery, West China Hospital, West China Medical School, Sichuan University, Chengdu, 610041 P. R. China; 5 Department of Medical Oncology, Cancer Center, West China Hospital, Sichuan University, Chengdu, 610041 P. R. China

**Keywords:** Tumour biomarkers, Predictive markers, Predictive markers

## Abstract

Phosphoglycerate kinase 1 (PGK1) has complicated and multiple functions in cancer occurrence, tumor progression and drug resistance. Sorafenib is the first-line treatment targeted drug for patients with kidney renal clear cell carcinoma (KIRC) as a tyrosine kinase inhibitor, but sorafenib resistance is extremely common to retard therapy efficiency. So far, it is unclear whether and how PGK1 is involved in the pathogenesis and sorafenib resistance of KIRC. Herein, the molecular mechanisms of PGK1-mediated KIRC progression and sorafenib resistance have been explored by comprehensively integrative studies using biochemical approaches, mass spectrometry (MS) identification, microarray assay, nude mouse xenograft model and bioinformatics analysis. We have confirmed PGK1 is specifically upregulated in KIRC based on the transcriptome data generated by our own gene chip experiment, proteomics identification and the bioinformatics analysis for five online transcriptome datasets, and PGK1 upregulation in tumor tissues and serum is indicative with poor prognosis of KIRC patients. In the KIRC tissues, a high expression of PGK1 is often accompanied with an increase of glycolysis-related enzymes and CXCR4. PGK1 exhibits pro-tumorigenic properties in vitro and in a xenograft tumor model by accelerating glycolysis and inducing CXCR4-mediated phosphorylation of AKT and ERK. Moreover, PGK1 promotes sorafenib resistance via increasing CXCR4-mediated ERK phosphorylation. In conclusion, PGK1-invovled metabolic reprogramming and activation of CXCR4/ERK signaling pathway contributes to tumor growth and sorafenib resistance of KIRC.

## Introduction

In recent years, the incidence of kidney cancer has been increasing year by year, reaching to proximate 2.2% of all new cancer cases and 1.8% of the total cancer deaths [1]. The most common form of kidney cancer is renal cell carcinoma (RCC) that arises from the renal epithelium. Although the development of diagnosis and treatment technology has prolonged the survival of RCC patients in recent years, tumor metastasis and chemoresistance still remain the main limiting factors for prognosis of patients. Nearly 25–30% of RCC patients present with a locally advanced or metastatic stage at the time of initial diagnosis [2].

Kidney renal clear cell carcinoma (KIRC) is the most common and fatal subtype of RCC, accounting for ~70–75% of RCC, and the other two subtypes including kidney chromophobe cell carcinoma and kidney renal papillary cell carcinoma account for about 25% of the remaining RCC [3]. Patients with KIRC have an extremely poor prognosis owing to the metastatic spread and radiochemoresistance [4]. KIRC is a highly vascularized malignant tumor, and several anti-angiogenesis tyrosine kinase inhibitors (TKIs), such as sorafenib, exhibit initially effective in tumor regression, but the acquired drug resistance will occur gradually and render sorafenib treatment ineffective for some patients [5]. Therefore, exploring new therapeutic targets and strategies to impede tumor progression and drug resistance are urgently needed concerns for RCC therapy.

Mutations of the von Hippel-Lindau (VHL) tumor suppressor gene have been observed in approximately 80% of KIRC tumors, which is one of the important factors driving the occurrence and development of KIRC [6]. VHL is a component of the E3 ubiquitin ligase complex to act as a negative regulator of hypoxia-inducible factor (HIF) signaling by targeting HIF-1/2α [7]. Missense mutation or inactivation of VHL in KIRC protects HIF-1/2α from VHL-mediated ubiquitination and degradation. Accumulated HIF-1/2α promotes the transcriptional activation of downstream target genes related to metabolism and angiogenesis.

Phosphoglycerate kinase 1 (PGK1) is a critical enzyme in the aerobic glycolysis process, which catalyzes the reversible transfer of a phosphate group from 1,3-bisphosphoglycerate to ADP, producing 3-phosphoglycerate and ATP to meet the energy needs of cells [8]. Several studies have demonstrated PGK1 is directly regulated by HIF-1α in many cancers [9–11]. PGK1 has a complicated and double-edged function as an oncogene or tumor suppressor in cancer occurrence and progression [12]. PGK1 and pyruvate kinase M2 (PKM2) are the only two enzymes that control ATP production during aerobic glycolysis in cells [13]. Indeed, the enzyme activity of PKM2 in cancer cells is very low, so PGK1 is probably more important than other glycolysis related enzymes for tumorigenesis [14]. By now only one literature has briefly mentioned MYC and its target gene PGK1 are up-regulated in RCC tissues [15]. So far, PGK1 functions in KIRC tumorigenesis and progression remain poorly understood. PGK1-mediated cell biochemical activities and sorafenib resistance have not been explored in renal cancer cells to date.

In this study, based on our gene chip and KIRC tissue microarray analyses, proteome MS identification and gene expression database exploring, we have uncovered PGK1 is highly increased in the tumor tissue and serum of KIRC patients, which is indicative of the clinical features of KIRC progression and prognostic value of PGK1. Furthermore, series of in vitro and in vivo experiments are performed to confirm PGK1-mediated tumorigenic processes and sorafenib resistance. In general, PGK1 activates the CXCR4/ERK pathway and accelerates glycolysis to enhance cancer progression and sorafenib resistance in KIRC cells.

## Materials and methods

### Cell culture

Human KIRC cell lines (786-O, OS-RC-2) [16, 17] were cultured in RPMI 1640 medium (Hyclone), and human KIRC cell line ACHN was cultured in DMEM medium (Hyclone). HK-2 is a human normal renal tubular epithelial cell line [18] cultured in F-12 medium (Hyclone). All the complete culture medium contained 10% fetal bovine serum (FBS, Gibco), 100 U/ml penicillin and 100 U/ml streptomycin (Beyotime Biotechnology). The authentication of these four cell lines was performed via short tandem repeat DNA profiling by the Feiouer Biotech Company (Chengdu, China).

The establishment of sorafenib-resistant 786-O cells (786-O^-R^) was referred to the previous reports [19]. Briefly, 786-O cells were incubated with sorafenib at a concentration just below the half maximal inhibitory concentration (IC_50_), and then the concentration of sorafenib was gradually increased by 0.5 μM every two passages. When sorafenib level reached 8 μM, the cells were further incubated for >10 passages before use in subsequent experiments.

### Biological or chemical reagents

Sorafenib (CAS #284461–73–0, purity: 99.92%), WZB117 (CAS #1223397–11–2, purity: 99.97%), and Plerixafor (also known as AMD3100) (CAS #110078–46–1, purity: >98.0%) were purchased from Chemexpress Inc (Shanghai, China).

### Lentivirus transfection and siRNA

To overexpress PGK1, the coding sequences of human PGK1 (RefSeq NM_000291.4) with a 3×Flag tag were cloned into *Xho*I and *Not*I restriction endonuclease sites of the FV115 lentiviral vector (Heyuan Biotechnology, Shanghai, China). This plasmid contains the coding sequences of PGK1 driven by human cytomegalovirus promoter, two 3ʹ long terminal repeats (LTR) genes derived from human immunodeficiency virus-1 (HIV-1), SV40 polyadenylation signal sequences and internal ribosome entry site (IRES) sequences of the encephalomyocarditis virus (EMCV) and some other essential elements.

ShRNA sequence targeting PGK1 (shPGK1) (5ʹ-CCGGCTGACAAGTTTGATGAGAATGCTCGAGCATTCTCATCAAACTTGTCAGTTTTTG-3ʹ) and a control non-targeting shRNA sequence (5ʹ-CCGGTTCCTGGAACAATTGCTTTTACTCGAGTAAAAGCAATTGTTCCAGGAATTTTTG-3ʹ) (shNC) were separately cloned into *Bam*HI and *Xho*I sites of lentiviral PLKO.1 vector (Tsingke, Beijing, China) [13]. The virus packaging and concentration were performed by Heyuan Biotechnology (Shanghai, China).

The PGK1-expressing plasmid or negative control (NC) vector was respectively transfected into 786-O, ACHN cells by lentiviral infection, and cells stably overexpressing PGK1 were screened and obtained under 2 μg/mL puromycin exposure for 7 days. 786-O cells with stably overexpressing PGK1 (786-O^-PGK1^) or empty vector (786-O^-NC^) were used to perform subsequent experiments.

Three siRNA sequences targeting PGK1 (siPGK1) were designed to determine the target one with best interference efficiency. siRNA transfection was performed using Lipofectamine 2000 (Invitrogen, USA).

(siPGK1#1) 5ʹ-GAGTCAATCTGCCACAGAA-3ʹ,

(siPGK1#2) 5ʹ-CCAAGTCGGTAGTCCTTAT-3ʹ,

(siPGK1#3) 5ʹ-GCATCAAATTCTGCTTGGA-3ʹ.

### Cell viability, colony formation and cell migration assay

Cell viability was detected by CCK-8 approach [17]. Each experiment was performed in triplicate. Stable transfected cells with density of 500 cells/well were seeded into a 6-well plate to culture for 10 d for colony formation assay. A transwell assay was conducted to evaluate cell migration ability [20]. The migratory cells were stained with crystal violet and images were acquired for cell counting under a microscope.

### Western blot

The specific primary antibodies against E-cadherin (ET1607–75), N-cadherin (ET1607–37), snail (ER1706–22), Flag (M1403–2), p-AKT(ET1607–73), AKT (ET1609–51), p-ERK (ET1610–13), ERK (ET1601–29), p-MEK (ET1609–50), CXCR4 (ER1802–28) and ENO1 (ET1705–56) were ordered from Hangzhou Hua-An Biotechnology Company. Other antibodies included β-actin (TA-09, ZSGB-BIO), MEK (sc-436, Santa Cruz), HK2 (22029–1-AP, Proteintech), ALDOA (11217–1-AP, Proteintech) and PGK1 antibody (sc-130335, Santa Cruz).

For serum PGK1 detection, the high abundance of serum albumin and IgG was deleted [21, 22] using a reagent kit following procedures (Albumin and IgG Erasin Kit, C500063–0005, Shanghai Sangon Biotechnology). Reversible Ponceau staining was used as a loading control.

The density of target band in Western blot was measured with Image J software (NIH, USA) for semi-quantification of staining signals.

### Tumor tissues and blood samples collected from KIRC

This research was approved by the Institutional Ethics Committee of State Key Laboratory of Biotherapy, West China Hospital of Sichuan University. Blood samples, tumor tissues and paired adjacent noncancerous tissues of KIRC patients were obtained from surgical specimens with informed consent at the Department of Urology, West China Hospital, Sichuan University. All diagnosis was confirmed by histopathological examination. Of these samples, 6 KIRC tissues and 6 paired adjacent noncancerous tissues were used for mRNA level detection by gene chip analysis, and the other cases were used for other researches.

A commercial tissue microarray with 90 KIRC tissues and 90 paired normal adjacent tissues (NATs) (HKid-CRC180Sur-01) was ordered from Shanghai Outdo Biotech Co., Ltd (Shanghai, China) to measure the protein expression of PGK1. The whole clinicopathological data included patient age, gender, tumor size, pathological grade, clinical stage, survival time, and follow-up data.

### Immunohistochemistry and immunofluorescence staining

Immunohistochemistry (IHC) staining and evaluation was carried out as we previously described [23]. The primary anti-PGK1 antibody (sc-130335, Santa Cruz) and anti-Ki67 antibody (ET1609–34, Hangzhou Hua-An Biotechnology) were diluted at 1:200 to perform IHC. Cells were plated and grown on coverslips overnight for cell immunofluorescence staining following reported approach [20].

### Gene chip analysis for mRNA level

Expression levels of mRNAs in 6 KIRC tissues and 6 paired normal adjacent tissues were detected on a Human GE 4×44 K v2 Microarray (G2519F-026652, Agilent Technologies). The clinical information of 6 KIRC species for gene chip analysis was provided in Supplementary Table 1. Flow chart of the gene chip experimental design was shown in Supplementary Fig. 1. In order to reduce the error caused by individual differences in patients, we mixed total RNA in pairs to detect. RNA samples were of high quality and reliability (Supplementary Table 2) for gene chip analysis (Human GE 4×44 K v2 Microarray). Agilent Technologies, Inc. was commissioned to perform labeling, array hybridization and scanning according to the standard protocol.

### Xenograft tumor model

Immunodeficient female BALB/c nude mice (five-week-old) were purchased from GemPharmatech Co.Ltd (Nanjing, China) to perform mouse xenograft experiments, which was approved and conducted by the Institutional Animal Care and Treatment Committee of Sichuan University. To study the effect of PGK1 overexpression on tumorigenicity of 786-O cells in vivo, 1×10^7^ of 786-O^-PGK1^ or 786-O^-NC^ cells were suspended in 150 µL of PBS to subcutaneously inoculate into each nude mouse. Similarly, 1×10^7^ of 786-O^-shPGK1^ or 786-O^-shNC^ cells were suspended in 150 µL of PBS to subcutaneously inoculate to each nude mouse to observe tumorigenicity under PGK1 knockdown.

The length (L) and width (W) of the xenograft tumors were measured every 3 days, with the W smaller than the L. The volume (V) of tumors was calculated as V = (L×W^2^)/2. Tumor nodules were collected for further examination.

### LC-MS/MS-based label-free quantitative proteomic identification

Protein extracts were isolated from each group of cells with RIPA buffer (P0013, Beyotime) containing 1 mM PMSF (ST506–2, Beyotime). To remove SDS detergent from the samples as well as to reduce and alkylate proteins, the filter-assisted sample preparation method (FASP) was performed subsequently [24]. Around 200 µg proteins were subjected to digestion following the FASP procedure. The resulting peptides were desalted using the spinnable StageTip protocol followed by nanoLC-MS/MS analysis using an Easynano-LC 1000 HPLC system (ThermoFisher Scientific, San Jose, CA, USA) and a Q-Exactive mass spectrometer (ThermoFisher Scientific, San Jose, CA, USA) [25]. Protein identification, quantitation, and bioinformatics analyses were searched using MaxQuant and Perseus software respectively.

### Glucose consumption and lactate production measurement

Glycolysis levels were evaluated by measuring glucose consumption and lactate production. The procedures were included as following. PGK1-overexpressing or PGK1-knockdown 786-O cells were seeded into 6-well plates at a same density of 1×10^6^ cells in 2.5 mL complete culture medium per well to culture for 24 h, then cell medium was collected to measure glucose consumption and lactate production level. At the same time, cells were collected to count to normalize the cell numbers between the experimental 786-O^-PGK1^ (786-O^-shPGK1^) and the control 786-O^-NC^ (786-O^-shNC^) cells, which aimed to exclude the disturbance caused by cell growth difference on the relative level of glucose consumption and lactate production.

The concentrations of glucose consumption and lactate production were measured using a glucose assay kit (E1010, Applygen) and a lactate detection kit (A019, Nanjing Jiancheng Biotech) respectively, which were normalized by the cell numbers as described previously [26, 27].

### RNA-sequencing and microarray datasets analyses

Gene levels of PGK1 in a large scale of KIRCs were analyzed from datasets of GEO and TCGA databases respectively. The transcriptomic data for KIRC cases including 530 KIRC tissues and 72 NATs were downloaded from TCGA database online (https://portal.gdc.cancer.gov/), meanwhile the clinical data of TCGA-KIRC patients were downloaded online (http://xena.ucsc.edu/). The cBioPortal web-based tool (www.cbioportal.org/) was used to obtain the information of VHL mutation.

The GEO database (http://www.ncbi.nlm.nih.gov/geo) is an open public database of gene transcriptional expression profile. Four sets of gene expression profiles, including GSE6344, GSE15641, GSE36895 and GSE66270, were collected in the GEO database. The GSE6344 data including 10 KIRC tissues and 10 NATs were obtained from the GPL96 platform (Array Affymetrix Human Genome U133A Array). The data of GSE15641 were obtained with the GPL96 platform (Affymetrix Human Genome U133A Array) and came from 32 KIRC tissues and 23 NATs. The data of GSE36895 were based on the GPL570 platform (Affymetrix Human Genome U133 Plus 2.0 Array) and collected from 29 KIRC tissues and 23 NATs. 14 KIRC tissues and 14 paired NATs were measured for the GSE66270 dataset (GPL570 platform, Affymetrix Human Genome U133 Plus 2.0 Array).

The gene expression matrix and the corresponding platform TXT files of target datasets were downloaded. The background correction of probe data, standardization, and summarization were performed by using R software (R 3.6.1, https://cran.r-project.org/) and related packages (http://www.bioconductor.org/) [28]. The differentially expressed genes (DEGs) between KIRC tissues and normal kidney tissues were identified according to the conditions of a log2 fold change |log2FC | > 0.75 and an adjusted *P* value < 0.05 [29]. These DEGs were evaluated by Student’s *t* test.

### Bioinformatics software

Gene set enrichment analysis (GSEA) is a computational method that assesses whether a priori defined a set of genes shows statistically significant, concordant differences between two biological states. The GSEA software (v.4.0.3) was applied to assay in our samples [30]. KIRC samples in the target dataset were divided into two groups by the median value of PGK1. The h.all.v7.1.symbols.gmt was selected as the reference gene set. The gene set permutations were performed 1000 times.

PGK1-involved in biological pathways were queried by FunRich software (version 3.1.3). GEPIA (http://gepia.cancer-pku.cn/) and Oncomine (http://www.oncomine.org/) databases were used to analyze the difference of PGK1 expression between KIRC and normal kidney tissues. Kaplan–Meier Plotter (http://kmplot.com/analysis/) database was used to assess the correlation between PGK1 expression and relapse-free survival in KIRC patients. The STRING online database (https://string-db.org/) and Cytoscape software were used to construct the coexpression network of overlapped DEGs and identify KIRC-related hub genes [31]. DAVID database (https://david.ncifcrf.gov/) was used to perform Gene Ontology (GO) functional and KEGG pathway enrichment analyses. Chord diagram for KEGG analysis was performed following our previous report [32].

### Statistical analysis

Statistical analysis was performed using SPSS software (version 22.0; SPSS Inc, Chicago, IL, USA) and GraphPad Prism 7.0 (GraphPad Software, San Diego, CA, USA). Data were presented as the mean ± standard deviation (SD) of at least three independent experiments. Kaplan–Meier method was used to assess patients’ survival outcome. The difference was evaluated using the two-tailed Student’s *t* test. Differences were considered significant at *P < 0.05, **P < 0.01 and ***P < 0.001.

## Results

### PGK1 is identified as one crucial DEG related to KIRC

**Fig. 1:**
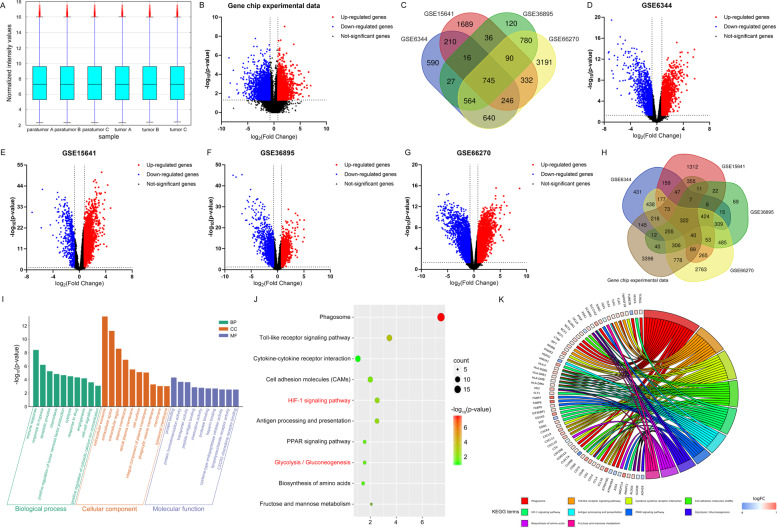
Differential gene expression profiling in KIRC based on gene chip experiment results and online GEO database. **A** Box plot of all the tested samples in the dataset after normalization. All six samples in the dataset were normalized, and the distributions of the log2-ratios were nearly same. **B** The volcano map of DEGs which were identified in KIRC from our microarray experiment results. **C** The 745 common DEGs were obtained among the four online datasets by the Venn diagram analysis. **D**–**G** The volcano map of DEGs identified from four online datasets including GSE6344, GSE15641, GSE36895, and GSE66270. The red points represented upregulated DEGs screened on the basis of fold change >0.75 and a corrected *P* value of <0.05. The blue points represented downregulated DEGs with a fold change < −0.75 and *P* value <0.05. The black points represented genes with no significant difference. **H** 322 crucial DEGs related to KIRC were selected by taking intersection of DEGs from four online GEO datasets (GSE6344, GSE15641, GSE36895, and GSE66270) and our gene chip experiment results. **I** GO functional analyses were performed to investigate the biological processes (BP), cellular component (CC), and molecular function (MF) terms enriched in 322 crucial DEGs related to KIRC. **J** DEGs were significantly enriched in HIF-1 signaling pathway and glycolysis among the top ten most significantly enriched KEGG pathways. **K** Chord diagram for KEGG analysis revealed that several PGK1-related signaling pathways were indicated to aberrantly alter in KIRC.

Firstly, we conducted a gene chip experiment (Supplementary Fig. 1) to observe transcriptome changes between KIRC and NATs. The gene chip dataset for differential gene analysis were normalized to make gene expression comparable across samples (Fig. 1A). The differences in gene expression between KIRC tissues and paired NATs were visualized by Volcano plots (Fig. 1B). With a threshold defined by a | log2FC | >0.75 and an adjusted P value < 0.05, a total of 6092 DEGs were identified from gene chip experiment results, including 3082 downregulated genes (log2FC < 0.75) and 3010 up-regulated genes (log2FC > 0.75) in the KIRC tissues. Gene expression profile obtained from our gene chip experiment was provided with detail in Supplementary Table 3.

To further validate the reliability of our gene chip experiment results, we verified the DEGs between KIRC tissues and NATs by exploring the gene profiling data from four KIRC-related GEO datasets. With a threshold defined by a | log2FC | >0.75 and an adjusted *P* value < 0.05, a total of 3038, 3364, 2378, and 6588 DEGs were extracted from the GSE6344, GSE15641, GSE36895, and GSE66270 datasets respectively. A total of 745 common DEGs were detected in the four datasets by using Venn diagram (Fig. 1C), and the volcano map of DEGs identified from each dataset was shown in Fig. 1D–G, respectively.

By taking intersection of DEGs obtained from our gene chip experiment results and four online GEO datasets, a total of 322 genes were defined as the crucial DEGs related to KIRC (Fig. 1H). Among these 322 genes, functions of majority have been investigated in KIRC, such as ATP1A1 and STC2 [17, 33], demonstrating the precision of our method for screening DEGs. Importantly, PGK1 is one object of the 322 key differential genes we screened. GO functional and KEGG analyses were further performed to investigate the biological processes (BP), cellular component (CC), molecular function (MF) and pathways enriched in 322 crucial DEGs related to KIRC. The top ten most significantly enriched GO terms in the BP, MF, and CC categories (Fig. 1I) and KEGG pathways (Fig. 1J) were assayed according to the integrative gene expression profiles. In fact, several important PGK1-relative biological processes and pathways are enriched, such as HIF-1 signaling pathway and glycolysis/ gluconeogenesis (Fig. 1J) that have been demonstrated playing an important role in occurrence and development of KIRC. Several PGK1-related signaling pathways are indicated to aberrantly alter in KIRC (Fig. 1K). So far, associations of PGK1 with KIRC still remain poorly understood. Considering the importance and complicated role of PGK1 in tumorigenesis, PGK1 attracts our strong interest to uncover its contribution to KIRC progression.

### PGK1 gene is specifically upregulated in KIRC

**Fig. 2:**
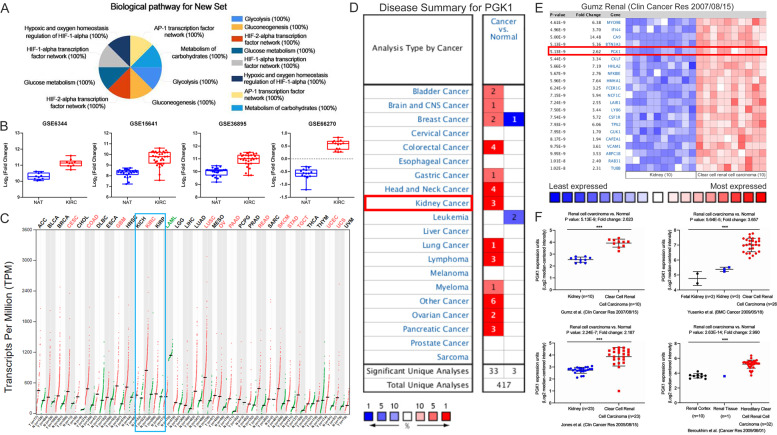
PGK1 is specifically upregulated in KIRC subtype of RCC. **A** PGK1-mediated biological pathways analyzed by FunRich software. **B** The expression levels of PGK1 gene were compared between KIRC tissues and NATs from online 4 datasets (GSE6344, GSE15641, GSE36895, and GSE66270). **C** PGK1 gene expression profile across all tumor samples and paired normal tissues from online GEPIA database. Each dot represented expression of a sample. **D** Expression pattern of PGK1 gene in multiple human malignancies assayed from the Oncomine database. The number in the colored cell represented the cases that satisfied the thresholds. The more intense red (overexpression) or blue (underexpression) suggested a more highly significant upregulated or downregulated gene. **E** The fold changes of mRNA levels of PGK1 in KIRC tissues compared with NATs from the Oncomine dataset (Gumz renal). **F** PGK1 gene was confirmed to significantly upregulate in KIRC from Oncomine database dissection. Oncomine datasets include 4 subtypes, including 1, Gumz renal; 2, Yusenko renal; 3, Jones renal; and 4. Beroukhim renal.

PGK1-mediated biological pathways were queried by FunRich software, which still demonstrated PGK1 is mainly involved in HIF1/2α transcription factor network and glucose metabolism (Fig. 2A). Importantly, these pathways are abnormally activated in KIRC. We then compared PGK1 gene expression levels in KIRC tissues and NATs based on the four online GEO datasets. Notably, the expression of PGK1 in KIRC tissues was significantly higher than that in NATs in all four datasets analysed. According to the transcriptome data from GSE6344, GSE15641, GSE36895 and GSE66270 datasets, PGK1 gene expression levels in KIRC samples were 1.79-, 1.77-, 2.62-, 1.89-, and 2.17-fold of those in corresponding NAT samples, respectively (Fig. 2B).

We further assayed PGK1 expression level in pathological subgroups of the kidney cancer. Indeed, PGK1 was specifically overexpressed in KIRC, but not in kidney chromophobe cell carcinoma (KICH) or kidney renal papillary cell carcinoma (KIRP) (Fig. 2C) when PGK1 expression profiling was assessed across all kinds of tumor tissue samples from GEPIA database. Moreover, PGK1 expression patterns in multiple human malignancies were compared from the Oncomine database. As expected, PGK1 level between cancer and normal tissues showed significant statistical differences in totally 36 separate analyses on multiple types of cancers, among which 3 sets of kidney cancer data confirmed PGK1 in kidney tumors was higher than that in corresponding normal tissues (Fig. 2D). Data from the Oncomine database also supported PGK1 was significantly upregulated in KIRC (Fig. 2E, F). The Oncomine data sets contained 4 subtypes, including Gumz renal, Yusenko renal, Jones renal and Beroukhim renal.

### PGK1 regulates glycolysis-related enzymes in KIRC cells

**Fig. 3:**
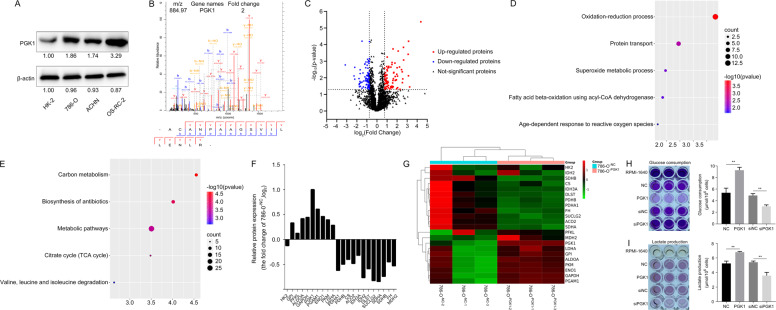
Comparison of proteomic profiles between KIRC cells with stably expressing PGK1 and mock transfection cells by LC-MS/MS quantification. **A** The endogenous protein levels of PGK1 were detected in 3 human KIRC cell lines and renal tubular epithelial HK-2 cell line. **B** MS/MS spectrum of PGK1 was presented. **C** DEPs were identified from 786-O^-PGK1^ cells compared to 786-O^-NC^ cells. **D**, **E** DEPs were involved in the top five most significantly biological processes and pathways. **F**, **G** PGK1-mediated expression changes of enzymes related to glycolysis and TCA cycle in 786-O cells. **H**, **I** PGK1 overexpression (suppression) significantly enhanced (decreased) glucose consumption and lactate production in KIRC cells.

To gain a comprehensive understanding of PGK1-regulated signaling pathways and biological processes in KIRC cells, we compared the proteomic expression differences between KIRC cells stably expressing PGK1 and mock cells with stably expressing empty vector by LC-MS/MS based on label-free quantification (LFQ) technology. Firstly, we detected the endogenous expression of PGK1 in 3 RCC cell lines. Compared with human normal renal tubular epithelial cell HK-2, protein level of PGK1 was relatively higher in 3 human RCC cells lines including 786-O, ACHN and OS-RC-2 (Fig. 3A). Among three RCC cell lines, PGK1 expression level was the highest in OS-RC-2, relatively lower in 786-O cells and the lowest in ACHN cells.

Since we focused on the function of PGK1 in cancer cell activity, we chose to overexpress PGK1 in 786-O and ACHN cells with relatively low expression of PGK1, and knock down PGK1 in OS-RC-2 cells that had a relatively high expression of PGK1 to perform follow-up analysis.

Total cellular proteins were extracted from 786-O^-PGK1^ cells and 786-O^-NC^ cells and digested into peptides for LC-MS/MS identification. PGK1 expression abundance was increased up to twice in 786-O^-PGK1^ cell lines compared with 786-O^-NC^ cells, and the MS/MS spectrum of PGK1 was presented in Fig. 3B. With a threshold defined by a | log2FC | >0.75 and an adjusted *P* value < 0.05, a total of 165 differential expression proteins (DEPs) between 786-O^-PGK1^ cells and 786-O^-NC^ cells were identified, including 78 upregulated DEPs and 87 downregulated DEPs (Fig. 3C), which were summarized in detail in Supplementary Table 4. PGK1-related DEPs were enriched in several biological processes and pathways, including oxidation-reduction process, protein transport, superoxide metabolic process (Fig. 3D), carbon metabolism, biosynthesis of antibiotics and metabolic pathways (Fig. 3E). These findings indicate that PGK1 has a great influence on the metabolic pathway of KIRC cells.

Particularly, PGK1 overexpression enhanced the expression of glycolysis-related enzymes and reduced tricarboxylic acid cycle (TCA cycle)-related enzymes in 786-O cells (Fig. 3F, G). The assay of glucose consumption and lactate levels in the supernatant of cultured KIRC cells was normalized based on the cell numbers [26, 27], which showed the glucose consumption (Fig. 3H) and lactate production were improved in 786-O^-PGK1^ cells (Fig. 3I) due to PGK1 overexpression when compared with the 786-O^-NC^ cells. Similarly, silencing PGK1 expression in 786-O cells significantly inhibited glucose consumption and lactate production (Fig. 3H, I). These results suggest PGK1 upregulation leads to expression changes of key enzymes in glycolysis and TCA cycle, then enhances glycolysis, and induces metabolic reprogramming to rapidly provide energy and nutriment for maintaining high speed of cell proliferation of KIRC cells.

### Upregulation of tissue/serum PGK1 is associated with poor prognosis for KIRC

**Fig. 4:**
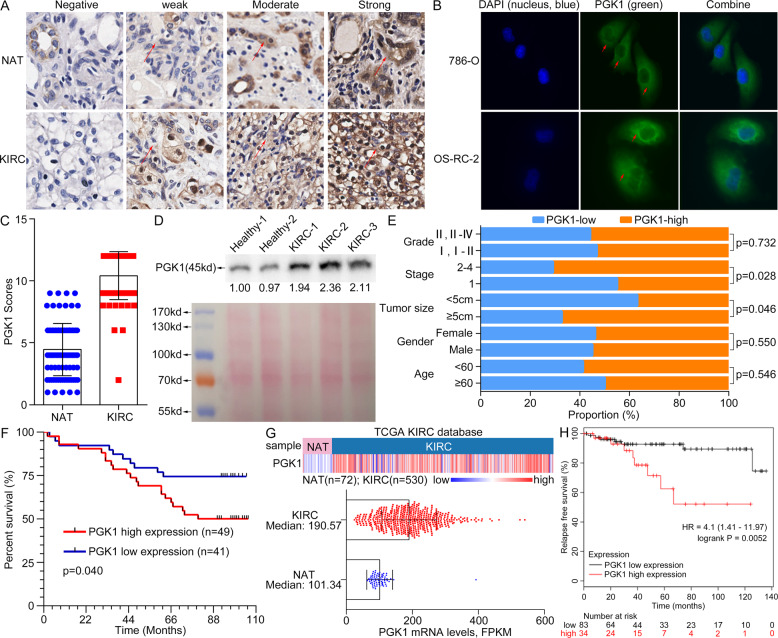
PGK1 upregulation in tumor tissues and serum is indicative of poor prognosis for KIRC patients. **A** Different staining level for PGK1 protein was shown in a paired noncancerous/tumor tissue from a commercial tissue microarray. Red arrows indicated positive staining for PGK1. **B** Cell endogenous PGK1 was mainly located in cytoplasm by cell fluorescence analysis. Red arrows meant positive fluorescent staining. **C** PGK1 level was increased in KIRC tissues by quantitative analysis of 90 cases of NATs and KIRC tissues. **D** The serum PGK1 was increased in KIRC patients compared to healthy persons by Western blot detection. Reversible Ponceau staining was used as a total loading control. **E** Associations of PGK1 levels and pathologic variables including grade, stage, tumor size, gender, and age obtained from tissue microarray. PGK1 protein level is well correlated with tumor size and tumor stage of KIRC (*P* < 0.05). **F** Kaplan–Meier overall survival curves for KIRC patients based on PGK1 expression from tissue microarray results. **G** Heatmap (top chart) and boxplot (bottom chart) for the transcriptional profiling of PGK1 in KIRC tissues (*n* = 530) and NATs (*n* = 72) respectively, derived from TCGA-KIRC species. **H** KIRC patients with high PGK1 mRNA level showed a shorter relapse-free survival (*P* < 0.05) from the KM-plotter database analysis.

We further investigated associations of PGK1 level and its clinicopathologic significance for KIRC patients. PGK1 protein expression profiling was validated by IHC on a commercial tissue microarray containing 90 KIRC tissues and 90 paired NATs (HKid-CRC180Sur-01, Outdo Biotech Co., Ltd.). The IHC staining of PGK1 protein in KIRC was defined 4 levels including negative, weak, moderate and strong expression (Fig. 4A). The IHC scores and clinicopathological data of these 90 samples were provided with detail in Supplementary Table 5. Tissue PGK1 was mainly distributed in cytoplasm. It was consistent with cell immunofluorescence staining in OS-RC-2 and 786-O cells (Fig. 4B). Moreover, the fluorescence intensity of PGK1 in OS-RC-2 cells was stronger than that in 786-O cells (Fig. 4B).

The average IHC score of PGK1 in 90 KIRC tissues was significantly higher than that in 90 paired NATs (10.42 ± 1.93 vs. 4.82 ± 0.41, *P* < 0.001) (Fig. 4C), and the proportion of strong staining of PGK1 in KIRC tissues was significantly higher compared with NATs (Table 1). Meanwhile, the secreted PGK1 into blood from KIRC cases was significantly elevated compared with healthy controls (Fig. 4D). KIRC cases were grouped into a high-PGK1 group and a low-PGK1 one according to the median IHC scores for tissue PGK1 to evaluate prognostic significance of PGK1 for patients. Generally, PGK1 protein level was closely related to tumor size and stage (*P* < 0.05) (Fig. 4E and Table 2), but not to age, gender and grade of KIRC patients. Moreover, the overall survival (OS) rate for patients (*n* = 49) with high expression of PGK1 was significantly lower than the low expression of PGK1 group (*n* = 41) (*P* < 0.05) (Fig. 4F).

**Table 1:** Tissue microarray assay of PGK1 expression in kidney renal clear cell carcinoma (No. HKid-CRC180Sur-01, Outdo Biotech).

Immunoreactivity	KIRC^a^ (*n* = 90)			NAT^b^ (*n* = 90)		
	Percentage	Total	Average^e^	Percentage	Total	Average
Weak^c^	1.11% (1/90)	2	2	32.22% (29/90)	62	2.14 ± 0.69
Moderate^c^	2.22% (2/90)	12	6	56.67% (51/90)	256	5.02 ± 1.01
Slightly strong^c^	42.22% (38/90)	336	8.84 ± 0.37	11.11% (10/90)	84	8.40 ± 0.52
Strong^c^	54.44% (49/90)	588	12	0% (0/0)	0	0
Total***^d^	100% (90/90)	938	10.42 ± 1.93	100% (90/90)	402	4.47 ± 2.11

^a^KIRC: kidney renal clear cell carcinoma.

^b^NAT: normal adjacent tissue.

^c^The weak, moderate, slightly strong and strong PGK1 levels were scored 1–3, 4–6, 7–9, 10–12, respectively.

^d^The immunoreactivity differences between RCC and NAT groups were estimated using Student’s *t* test, ***P < 0.001.

^e^Experimental values are represented as means ± SD.

**Table 2:** Tissue microarray analysis of correlation between PGK1 expression and clinicopathological parameters (No. HKid-CRC180Sur-01, Outdo Biotech).

Clinicopathological parameters	All patient number (n)	Expression level of PGK1 (*n* = 90)			
		High Number (n)	Low Number (n)	Average score^b^	*P* value^c^
Total	90	49	41	10.42 ± 1.93	
Age (years)					
<60	46	27	19	10.54 ± 2.03	0.546
≥60	44	22	22	10.30 ± 1.84	
Gender					
Male	51	28	23	10.53 ± 1.70	0.550
Female	39	21	18	10.28 ± 2.21	
Tumor size					
≥5 cm	52	35	17	10.77 ± 2.04	0.046
<5 cm	38	14	24	9.95 ± 1.68	
Stage					
1	60	27	33	10.05 ± 2.05	0.028
2	18	13	5	11.06 ± 1.49	
3	4	2	2	10.50 ± 1.73	
4	2	2	0	12	
unknow	6	5	1	11.50 ± 1.22	
Grade					
I	33	18	15	10.48 ± 1.77	0.732
I-II	14	7	7	10.50 ± 1.56	
II	28	15	13	10.14 ± 2.43	
II-III	4	4	0	12	
III	10	5	5	10.40 ± 1.71	
III-IV	1	0	1	9	

^a^The high- and low-level groups was determined by the average score of PGK1.

^b^Experimental values are represented as means ± SD.

^c^Differences between two groups (Age: <60 vs. ≥60; Gender: Female vs. Male; Tumor size: <5 cm vs. ≥5 cm; Stage: 1 vs. 2–4; Grade: I, I-II vs. II, II-IV) were assessed using the Student’s *t* test.

We further validated PGK1 association with patient’s poor prognosis in a large scale of KIRC samples by investigating the transcriptome and clinical data derived from TCGA dataset which included 72 NATs and 530 KIRC tissues. As results, PGK1 mRNA levels in KIRC tissues with a median value of 190.57 were significantly upregulated than those of NATs with a median value of 101.34 from TCGA-KIRC dataset analysis (Fig. 4G). The correlation between PGK1 level and survival time of KIRC patients was further evaluated by Kaplan–Meier Plotter tool, which also supported KIRC patients with high PGK1 mRNA had a shorter relapse-free survival (RFS) (*P* < 0.01) (Fig. 4H).

In conclusion, upregulation of tissue/serum PGK1 is indicative of short survival time and poor prognosis for KIRC patients.

### PGK1 induces a pro-tumorigenic phenotype in vitro and in vivo

**Fig. 5:**
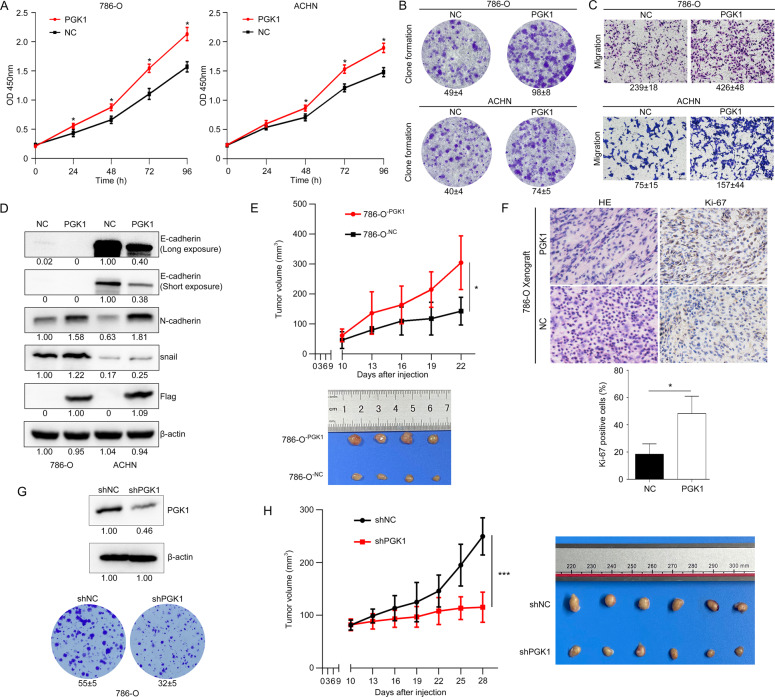
PGK1 induces a pro-tumorigenic phenotype in KIRC cells both in vitro and in vivo. PGK1 improved cell proliferation (**A**), clonogenic capacity (**B**) and migration (**C**) in 786-O and ACHN cells. Data were presented as the mean ± SD. **D** The epithelial-mesenchymal transition (EMT) marker proteins were detected in the PGK1-overexpressing RCC cells. **E** PGK1 overexpression enhanced tumor growth of xenograft mice. Upper panel: tumor growth curve of xenograft mice during inoculation period. 786-O cells stably overexpressing PGK1 or control plasmid were injected subcutaneously into BALB/c nude mice (*n* = 4), and tumor sizes and morphology were evaluated 5 times after injection. * *P* < 0.05. Bottom panel: Representative images of xenograft tumors at day 22 after subcutaneous cell injection. **F** Comparison of Ki-67 staining between 786-O^-PGK1^ and 786-O^-NC^ cells. **G**, **H** Knockdown of PGK1 expression inhibited the cell clonogenic capacity in vitro and the tumorigenicity of 786-O cells in nude mice.

PGK1-mediated cell biological behaviors were observed in 786-O^-PGK1^ and ACHN^-PGK1^ cells. The exogenous expression of PGK1 significantly enhanced KIRC cell proliferation (Fig. 5A). At the same time, PGK1 promoted cell clonogenic capacity (Fig. 5B) and migration (Fig. 5C) of 786-O and ACHN. More importantly, biomarkers of epithelial-mesenchymal transition (EMT), such as N-cadherin and snail, were significantly increased, but E-cadherin was obviously decreased under PGK1 induction (Fig. 5D). The expression level of E-cadherin in 786-O cells was extremely low and weakly visible only under a long western blot exposure, while it was nearly undetectable under a short western blot exposure.

We further constructed a xenograft tumor model by injecting 786-O^-PGK1^ cells and 786-O^-NC^ cells to investigate PGK1 influences on carcinogenesis of KIRC in vivo. The tumor was observed at day 10 after injection of 786-O cells in nude mice. Then at day 22 after subcutaneous injection of 786-O cells, the xenograft tumors were peeled from the euthanized mice to detect PGK1 expression level and relative proteins. The tumor volume inoculated with 786-O^-PGK1^ cells was bigger than that of being inoculated with the mock 786-O^-NC^ cells after 13 days of cell inoculation (Fig. 5E). And at 22 days of inoculation, the tumor volume difference was the greatest, 304.19 ± 89.63 mm^3^ vs. 142.49 ± 40.16 mm^3^ (*P* < 0.05). Moreover, more intense Ki-67 staining was observed in xenograft tumor inoculated 786-O^-PGK1^ cells compared the mouse tumor inoculated 786-O^-NC^ cells (Fig. 5F). These data indicated 786-O^-PGK1^ cells exhibited a stronger proliferative capacity than 786-O^-NC^ cells, and PGK1 upregulation contributed to tumorigenicity of KIRC in vivo.

Meanwhile, we established a xenograft nude mouse model to observe PGK1-knockdown influence on tumor growth. The lentivirus packaged shRNA targeting PGK1 (shPGK1) was transfected to 786-O cells to generate stable PGK1-knockdown cells. The stable knockdown of PGK1 expression significantly inhibited the cell clonogenic capacity in vitro (Fig. 5G) and the tumorigenicity of 786-O cells in nude mice (Fig. 5H), which further strengthened our conclusion that PGK1 induces a pro-tumorigenic phenotype in KIRC cells in vivo.

### PGK1 and CXCR4 synergistically promotes KIRC cell proliferation

**Fig. 6:**
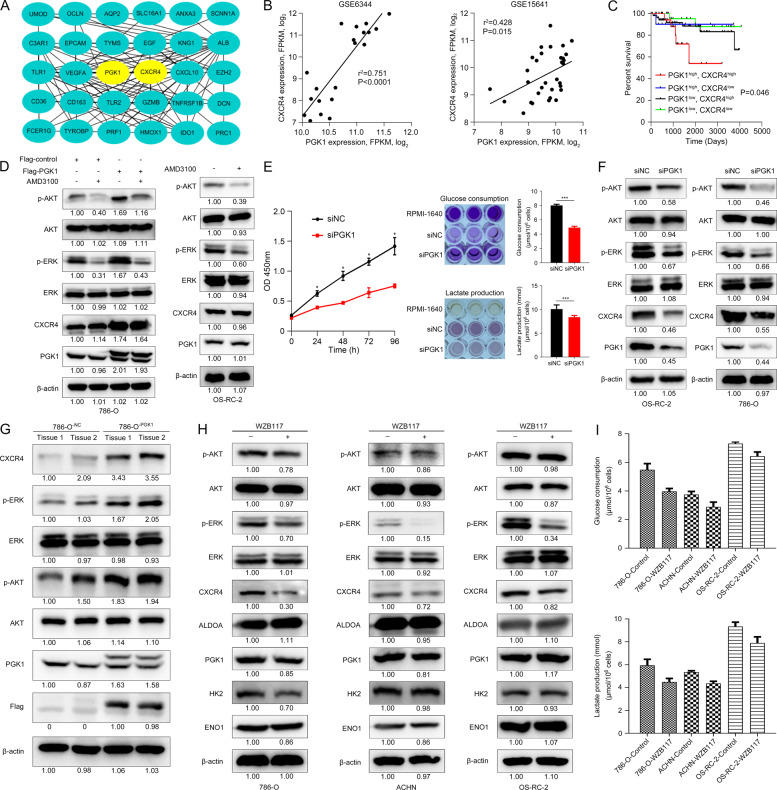
PGK1 activates CXCR4/ERK pathway in KIRC cells / tissues. **A** PGK1 and CXCR4 were the hub genes in KIRC, and there was a strong correlation between PGK1 and CXCR4. **B** Co-expression of PGK1 and CXCR4 transcript levels from GSE6344 and GSE15641 dataset. **C** Kaplan–Meier’s survival analysis of the correlation between the combination of PGK1 and CXCR4 expression levels and disease-free interval (DFI) survival from TCGA KIRC objects. **D** PGK1 induced the CXCR4-mediated phosphorylation of AKT (p-AKT) and ERK (p-ERK), and blockade of CXCR4 signaling by AMD3100 treatment did not alter cellular PGK1 expression in KIRC cells. **E** PGK1 knockdown inhibited cell proliferation and decreased glucose consumption and lactate production in OS-RC-2 cells. The darker the color develops, the more glucose remaining and lactate produced. **F** CXCR4, p-AKT, and p-ERK were decreased by PGK1 inhibition in 786-O and OS-RC-2 cells. **G** Protein levels of PGK1, CXCR4, p-AKT, and p-ERK were detected in xenograft tumors inoculating 786-O^-PGK1^ cells. β-actin served as the internal control. Glycolysis suppression by 40 μM WZB117 treatment for 48 h significantly downregulated CXCR4 and p-ERK (**H**) and reduced glucose consumption and lactate production (**I**) in RCC cells.

By importing the 322 overlapped DEGs into the online tool STRING [31], we constructed the protein-protein interaction (PPI) network of these genes. Further the interaction network data was imported into Cytoscape software to identify KIRC-related hub genes. The top 30 genes with the highest betweenness centrality were regarded as hub genes. Among the candidate genes, PGK1 and CXCR4 were the hub genes in KIRC, and there was a strong correlation between PGK1 and CXCR4 (Fig. 6A).

Furthermore, linear regression analysis revealed a positive correlation between PGK1 and CXCR4 mRNA in 10 KIRC samples and 10 NAT samples from GSE6344 dataset and 32 KIRC cases from GSE15641 dataset (Fig. 6B). The KIRC samples with high mRNA expression of PGK1 often owned high mRNA expression of CXCR4 correspondingly. Moreover, compared to prognosis prediction independently by PGK1 for KIRC patient survival, we found that the combination of PGK1 and CXCR4 seemed to be able to predict patient survival more accurately. KIRC patients with high expression of PGK1 and CXCR4 had the worst disease-free interval (DFI) survival (Fig. 6C). These results suggest that PGK1 and CXCR4 could be important biomarkers for prognosis of KIRC patients.

We further investigated the molecular mechanism of PGK1-CXCR4 involvement in KIRC cells. As known, activation of CXCR4 signaling triggered the downstream AKT and ERK signaling pathways [34]. Similarly, expression of CXCR4, p-AKT and p-ERK was increased in 786-O^-PGK1^ cells (Fig. 6D, left panel, band 3 vs band 1) when compared with the 786-O^-NC^ cells, which demonstrated PGK1 overexpression enhanced CXCR4, p-AKT and p-ERK levels.

On the contrary, when cells were treated CXCR4 antagonist AMD3100 for 48 h, the inhibition of p-AKT and p-ERK levels in 786-O^-PGK1^ cells was relatively weaker than that in 786-O^-NC^ cells (Fig. 6D, left panel, band 4 vs band 2), suggesting that PGK1 overexpression-induced CXCR4 upregulation contributed to compensate with the decrease of p-AKT and p-ERK caused by CXCR4 antagonist. However, blocking of CXCR4 signaling by AMD3100 treatment did not alter cellular PGK1 expression. In conclusion, our results demonstrated that PGK1 overexpression induced the CXCR4-mediated phosphorylation of AKT and ERK in RCC cells.

In OS-RC-2 cells with the highest expression of PGK1, we designed three siRNA sequences to interfere with the expression of PGK1. The siRNA sequence (siPGK1#1) with the best interference effect was used for subsequent experiments Knockdown of PGK1 inhibited cell proliferation by decreasing glucose consumption and lactate production in OS-RC-2 cells (Fig. 6E). Correspondingly, the expression of CXCR4 and p-AKT and p-ERK were downregulated by PGK1 inhibition in OS-RC-2 and 786-O cells (Fig. 6F). It was consistent that the expression levels of PGK1, CXCR4, p-AKT, p-ERK in xenograft tumor tissues were increased in 786-O^-PGK1^ group compared with the 786-O^-NC^ group (Fig. 6G). All results indicated that increase of p-AKT, p-ERK in KIRC cells was cooperation of PGK1 upregulation and the activation of CXCR4 signaling to a certain extent.

We further verify influence of glycolysis inhibition on cell CXCR4/ERK signaling. WZB117, a small molecule inhibitor of GLUT1 by downregulating glycolysis, has been shown to inhibit tumor growth [35]. We treated RCC cells with 40 μM WZB117 for 48 h to observe effects of glycolysis suppression on CXCR4/ERK signaling pathway. As a result, WZB117 led to a decrease of CXCR4, p-AKT and p-ERK in RCC cells (Fig. 6H). It was noticed that WZB117 led to a decrease of p-AKT in RCC cells with a different degree, and it was relatively obvious in 786-O cells. Generally, the inhibition degree of p-AKT by WZB117 was not as obvious as that of p-ERK. Although WZB117 treatment did not affect expression of PGK1 and other glycolysis-related enzymes, it significantly reduced the glucose consumption and lactate production of RCC cells (Fig. 6I). These results suggest PGK1-mediated CXCR4/ERK signaling is affected by glycolysis.

### PGK1 level is correlated with gene expression of glycolysis-related enzyme in KIRC

**Fig. 7:**
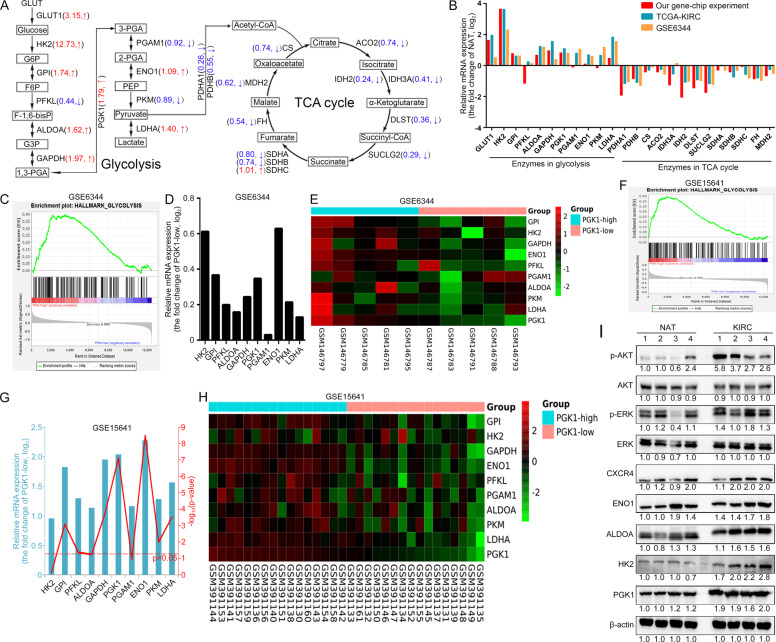
PGK1 is positively correlated with key glycolysis-related gene expression in KIRC clinical samples. **A** Schematic representation of differences in the mRNA expression levels of enzymes related to glucose metabolic pathways (glycolysis and TCA cycle) between normal kidney tissues and KIRC tissues. The values indicated the fold change over the normal sample (positive values represented upregulation compared with the normal sample, negative values represented downregulation compared with the normal sample). **B** Glucose metabolic relative gene expression profiling was summarized from our gene chip results combined with online transcriptome data from GSE6344 and TCGA-KIRC databases. PGK1 expression in KIRC was positively correlated with glycolysis process (**C**) including several glycolysis-related enzymes (**D**) according to GSE6344 dataset analysis. **E** Heat map of the glycolysis-related DEGs from high abundance of PGK1 group (*N* = 5) and low abundance of PGK1 group (*N* = 5) from GSE6344 dataset analysis. **F**, **G** GSE15641 dataset with the largest sample size demonstrated PGK1 upregulation was often accompanied by high expression of other glycolysis-related enzymes. **H** Heat map of the glycolysis-related DEGs from high abundance of PGK1 group (*N* = 16) and low abundance of PGK1 group (*N* = 16) from GSE15641 dataset analysis. **I** The protein expression levels of glycolysis-related enzymes, CXCR4, p-AKT, and p-ERK were shown in four randomly selected KIRC tissues and NATs.

As well-known, PGK1 is a glycolytic kinase that activates intracellular glycolysis to provide ATP for the rapid growth of cancer cells. We would like to further confirm whether this is one of the important mechanisms by which PGK1 regulates KIRC progression. By exploring the transcriptome data of our own gene chip experiment, we found that most of the glycolysis-related enzymes including GLUT1, HK2, GPI, ALDOA, GAPDH, ENO1 and LDHA were increased in KIRC, while those related to TCA cycle such as PDHA1, PDHB, CS, ACO2, IDH2, IDH3A, DLST, SUCLG2, FH and MDH2 were decreased in KIRC (Fig. 7A). The expression level of PGK1 in KIRC was 1.79 times of that in normal tissues.

In addition to our gene chip experiment results, the transcriptome data from online GSE6344 and TCGA-KIRC databases also supported our conclusion of glycolysis-related enzymes upregulated and TCA cycle-related enzymes downregulated in KIRC (Fig. 7B). The GSE6344 dataset is the most representative dataset for studying KIRC gene expression changes [36]. Through GSEA analysis of the transcriptome data in the GSE6344 dataset, we found that PGK1 expression was positively correlated with glycolysis process in KIRC (Fig. 7C). That is to say, the mRNA levels of glycolysis-related enzymes in samples with high expression level of PGK1 were higher than those with low expression of PGK1 (Fig. 7D, E). GSE15641 dataset with the largest sample size was selected for further validation, and a same conclusion was obtained as GSE6344 (Fig. 7F). In general, in KIRC tissues with high expression of PGK1, it is often accompanied by high level of other glycolysis-related enzymes (Fig. 7G, H). Moreover, the protein expression levels of glycolysis-related enzymes, CXCR4, p-AKT and p-ERK were overall higher in four randomly selected KIRC tissues compared with NATs.

In a word, considering gene expression associations of PGK1 with glycolysis-related enzymes, and PGK1 upregulation related to KIRC occurrence, we speculated PGK1-invovled glycolysis sustained tumor cell growth and survival for KIRC progression.

### PGK1 enhances sorafenib resistance via increasing ERK phosphorylation

**Fig. 8:**
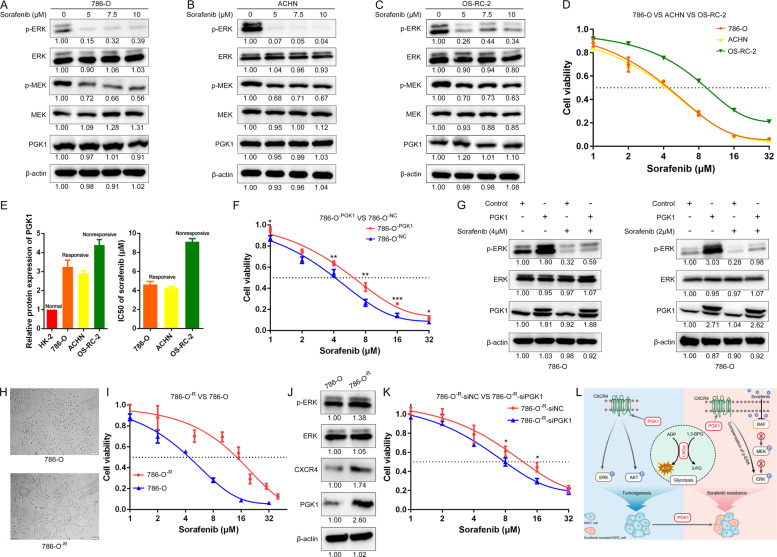
PGK1 promoted sorafenib resistance via PGK1/CXCR4/ERK signaling. **A**–**C** Sorafenib could inhibit Raf/MEK/ERK signaling pathway in all three KIRC cells. After treatment with sorafenib at the same concentration for the same time, the expression of residual phosphorylated ERK was the highest in OS-RC-2 cells. **D** CCK8 assays of three KIRC cells after sorafenib treatment at the indicated concentrations for 48 h. **E** Histograms showing the PGK1 protein expression level and IC_50_ values (48 h) of three KIRC cells. **F** CCK-8 assay of PGK1-overexpressing and control 786-O cells after sorafenib treatment at the indicated concentrations for 48 h. **G** PGK1 promoted sorafenib resistance via increasing ERK phosphorylation in 786-O cells. **H** Schematic diagram of morphological differences between sorafenib-resistant 786-O cells and wild-type 786-O cells. **I** CCK-8 assay of sorafenib-resistant 786-O cells after sorafenib treatment at the indicated concentrations for 48 h. **J** Expression levels of PGK1, CXCR4, ERK and its phosphorylation were detected in sorafenib-resistant 786-O cells and wild-type 786-O cells. **K** Interference with PGK1 expression in our constructed 786-O^-R^ cells increases their sensitivity to sorafenib. **L** Schematic diagram of the PGK1-invovled signaling pathway in KIRC progression and sorafenib resistance. In addition to promoting glycolysis to produce ATP for energy supply, PGK1 also induced CXCR4-mediated activation of AKT and ERK phosphorylation to promote tumor growth. Since the RAF/MEK/ERK signaling pathway is one typical target of sorafenib, PGK1 promoted sorafenib resistance via not only accelerating glycolysis but also compensating for the inhibition of ERK phosphorylation caused by sorafenib.

PGK1 has been proven to be able to promote the radiochemoresistance of some tumors due to its role in glycolysis activation [12]. However, whether PGK1 is related to the resistance of tumor cells to TKI drugs has not been reported. Sorafenib, as a TKI drug, is still the first-line treatment for advanced RCC [37]. Our experiments confirmed that sorafenib inhibited Raf/MEK/ERK signaling pathway in all three KIRC cells (Fig. 8A-C). Interestingly, after treatment with sorafenib at the same concentration for the same time, the expression of residual phosphorylated ERK was the highest in OS-RC-2 cells. In other words, the inhibitory effect of sorafenib on ERK phosphorylation in OS-RC-2 cells was not as effective as that on 786-O and ACHN cells, which indicates OS-RC-2 is more resistant to sorafenib.

Next, we examined the IC_50_ values of sorafenib in these three KIRC cell lines. Cell proliferation with several serial concentrations of sorafenib treatment for 48 h showed the average IC_50_ of sorafenib was 4.64, 4.25, and 9.11 μM for 786-O, ACHN, and OS-RC-2 cells respectively (Fig. 8D). Coincidentally, the IC_50_ values of sorafenib in these three KIRC cells were positively correlated with the protein expression of endogenous PGK1 (Fig. 8E). A preliminary conclusion is KIRC cells with higher endogenous PGK1 level have higher IC_50_ values for sorafenib. The IC_50_ value of sorafenib was increased from 4.22 μM in 786-O^-NC^ cells to 6.18 μM in 786-O^-PGK1^ cells (Fig. 8F). In general, PGK1 overexpression promotes sorafenib resistance in 786-O cells.

Upon serial concentrations (1, 4, 8, 16 and 32 µM) of sorafenib exposure, cell viability of 786-O^-PGK1^ cells was better than the 786-O^-NC^ cells (*P < 0.05, **P < 0.01 and ***P < 0.001 by two-tailed Student’s *t* test). The mean IC50 value of sorafenib was increased from 4.22 μM in 786-O^-NC^ to 6.18 μM in 786-O^-PGK1^ cells (Fig. 8F).

Upon 4 μM sorafenib treatment for 48 h, p-ERK level in 786-O^-PGK1^ cells was 1.79-fold of the control 786-O^-NC^ cells (Fig. 8G, left panel, band 4 vs band 3). Mechanistically, PGK1 overexpression was able to partially resist the inhibition of p-ERK by 4 μM sorafenib treatment in 786-O cells, but it was not sufficient to fully compensate to the inhibition of p-ERK by a relatively high concentration of 4 μM treatment for 48 h. Another experiment data confirmed that PGK1 overexpression could completely counteract the inhibitory effect on p-ERK due to a low concentration of 2 μM sorafenib exposure (Fig. 8G, right panel, band 4 vs band 1).

To further explore the molecular mechanism underlying sorafenib resistance in KIRC, we successfully induced and constructed sorafenib-resistant 786-O (786-O^-R^) cells which were derived from sorafenib-sensitive 786-O cell by constant sorafenib inducement. The morphology of 786-O^-R^ cells was flatter and longer than that of 786-O wild-type cells (Fig. 8H). The IC_50_ value of 786-O^-R^ cells was 15.07 μM, which was three times more than that of wild-type 786-O cells with 4.64 μM (Fig. 8I). In addition, the protein expression levels of PGK1, CXCR4 and p-ERK were increased in 786-O^-R^ cells (Fig. 8J), which indicates that PGK1 upregulation activates CXCR4/p-ERK signaling and contributes to sorafenib resistance.

Moreover, gene silencing of PGK1 resulted in a significant reduction in cell viability of 786-O^-R^ cells upon 8 or 16 μM sorafenib treatment (* indicates *P* < 0.05 by two-tailed Student’s *t* test) (Fig. 8K). The IC_50_ value of 786-O^-R^-siPGK1 cells was 7.88 μM, which was significantly lower than the IC_50_ value of 13.93 μM for 786-O^-R^-siNC cells.

## Discussion

In this study, we have confirmed that PGK1 is specifically overexpressed in KIRC tissues, not in KICH or KIRP tissues, which is of great significance for the accurate diagnosis and treatment of kidney cancer. To our knowledge, this is the first report to comprehensively investigate the gene expression, function and regulatory mechanism of PGK1 in RCC by integrating multiple data from bioinformatics analysis, cancer cell biology, nude mouse tumor model and clinical tumor tissue validations.

KIRC is the most common subtype of RCC that is strongly associated with alterations in the VHL gene. The loss or mutation of VHL made it impossible for VHL to degrade HIF-α by ubiquitin–proteasome pathway, which leads to the accumulation of HIF-α [38]. As a downstream target of HIF-α [9–11], PGK1 may be related to the loss or mutation of VHL. As we expected, in 530 TCGA-KIRC clinical samples, PGK1 expression was negatively correlated with VHL expression at the mRNA level (Supplementary Fig. 2A). Moreover, the VHL mutation is associated with PGK1 increase in KIRC tumors based on the analysis of TCGA cohort of KIRC tumors (Supplementary Fig. 2B). Apart from TCGA database, the clinical samples from GEO database also support the conclusion that PGK1 is negatively correlated with the expression of VHL at mRNA level (Supplementary Fig. 2C, D). In addition, through GSEA analysis of the transcriptome data from the GSE6344 dataset and GSE15641 dataset, we found that PGK1 expression was positively correlated with tumor hypoxia in KIRC (Supplementary Fig. 2E, F). Taken together, PGK1 upregulation is associated with VHL inhibition/mutation and tumor hypoxia in KIRC tissues. Our results partly explain the reasons that PGK1 is specifically overexpressed in KIRC tissues rather than in KIRP and KICH tissues.

At present, sorafenib is the first-line drug for RCC [37], but patients with advanced RCC resistant to sorafenib have limited clinical treatment options. So far, exploring sorafenib resistance mechanism in RCC is to find therapeutic targets for coping with this problem. As a downstream target of HIF-α, PGK1 can be activated under hypoxia [9–11], thus promoting the glycolysis process to produce a large amount of ATP for energy supply and resisting the adverse conditions brought by sorafenib. The lasting sorafenib treatment results in angiogenesis inhibition and intratumoral hypoxia, thereby inducing HIF-α expression [39]. There is a marked correlation between the hypoxic microenvironment and sorafenib resistance in hepatocarcinoma, and HIF-α-induced glycolysis activation is critical for sorafenib resistance in hepatocarcinoma [40, 41]. In our study, we found that either the transcriptome data from our own microarray experiment, the transcriptome data from the GEO database, or the transcriptome data from the TCGA database, all revealed a phenomenon that PGK1 gene expression was positively correlated with glycolysis-related enzyme gene expression in KIRC clinical samples. By analysing the gene expression data of KIRC tissues, we confirmed PGK1 is involved in the glycolysis process, which contributes to the tumor growth and sorafenib resistance in KIRC.

Sorafenib is a multikinase inhibitor with antiangiogenic activity by targeting VEGFR and PDGFR, and it also blocks Raf/MEK/ERK pathway to inhibit tumor growth [42]. The activation of Raf/MEK/ERK pathway has been confirmed to be very important for sorafenib resistance in RCC and hepatocarcinoma [5, 43]. It has been reported the activation of CXCR4 pathway increases the phosphorylation of AKT and ERK [34]. Several studies have demonstrated PGK1 can enhance the proliferation and migration ability of tumors by activating CXCR4 in gastric cancer and neuroblastoma [44–46], but PGK1/CXCR4/ERK molecules and related signaling pathways have not been investigated in RCC in previous reports. Interestingly, the loss or mutation of VHL can lead to overexpression of CXCR4 [47, 48]. Therefore, we speculated PGK1 and CXCR4 are closely related in KIRC progression.

Hub genes are genes that play crucial roles in biological processes. We constructed the coexpression network of overlapped DEGs to screen KIRC-related hub genes according to previous reports [49, 50], and the results showed that both PGK1 and CXCR4 are KIRC-related hub genes and highly expressed in KIRC tissues, and there is a strong interaction between PGK1 and CXCR4. The KIRC clinical samples with high mRNA level of PGK1 often own high mRNA expression of CXCR4 correspondingly.

We further confirmed PGK1 overexpression in 786-O cells activates CXCR4/ERK pathway to reverse the inhibition of ERK phosphorylation by sorafenib, which is one of the important mechanisms responsible for PGK1-mediated sorafenib resistance in KIRC. Similarly, PGK1, CXCR4 and ERK phosphorylation were significantly higher in the sorafenib-resistant 786-O cells by sorafenib-induced acquired resistance than those in wild-type 786-O cells. Our conclusion is consistent with another previous report of CXCR4 overexpression in the KIRC tissues from patients who are resistant to sorafenib [51]. Therefore, the mechanism on PGK1-mediated signaling pathway in KIRC progression and sorafenib resistance was summarized in Fig. 8L. Generally, PGK1 upregulation contributes to tumorigenesis and sorafenib resistance of renal clear cell carcinoma via activating CXCR4/ERK signaling pathway and accelerating glycolysis.

CXCR4 has been reported to relate with resistance to some TKI drugs, including sunitinib and sorafenib. Inhibition of CXCR4 significantly enhances sensitivity of hepatoma cells to sorafenib [52, 53], while high CXCR4 expression correlates with poor response upon sunitinib treatment for metastatic RCC [54, 55]. Therefore, a combination of PGK1 and CXCR4 will serve as a powerful prognostic factor for KIRC patients, which may provide novel molecular targets for the diagnosis and treatment of KIRC patients.

Recently, PGK1 has gradually become a hot topic in cancer research due to its involvement of a variety of biological activities [12]. PGK1 can be secreted into blood and detected in serum, and human serum PGK1 level is more conveniently monitored than tissue samples. Previous studies have confirmed that serum PGK1 levels in patients with liver cancer, pancreatic cancer and lung cancer are significantly higher than those in healthy controls [56–58]. Our studies also validate tumor tissue and serum PGK1 levels are associated with poor prognosis of KIRC patients. This indicates PGK1 has great potential to be a biomarker for monitoring KIRC progression and target drug treatment efficiency.

PGK1 exhibits pro-tumorigenic properties in a variety of tumors. Our study also confirmed that PGK1 played a critical role in KIRC occurence and sorafenib resistance. Therefore, the development of PGK1 inhibitors is very important. The combination therapy of PGK1 inhibitors with sorafenib will overcome the problem of drug resistance for KIRC treatment. Unfortunately, PGK1 inhibitors are still unavailable so far [13]. Currently, Terazosin and CBR-470–1 are small molecular compounds to potentially inhibit PGK1, but their efficiency in tumors remain unclear [59, 60]. The development of specific PGK1 inhibitors through virtual screening, compound modification even de novo synthesis and their application in the treatment of PGK1-related cancers are going on the way.

## Conclusions

In summary, our results indicate that PGK1 overexpression enhances tumorigenesis and sorafenib resistance of KIRC via activating the CXCR4/ERK signaling pathway and accelerating glycolysis (Fig. 8L). Moreover, our work provides strong evidences that PGK1 is an excellent diagnostic and prognosis marker, as well as a promising drug target for KIRC.

## Supplementary information

Supplementary Figure 1

Supplementary Figure 2

Supplementary legends

Dataset 1

Dataset 2

Dataset 3

Dataset 4

Dataset 5

Reproducibility checklist

## Data Availability

The authors declared that all the data and materials are available on reasonable request.
