# Supplementary figures and images for: PGK1 contributes to tumorigenesis and sorafenib resistance of renal clear cell carcinoma via activating CXCR4/ERK signaling pathway and accelerating glycolysis
Source: Cell Death Dis. 2022 Feb 4;13(2):118. doi: 10.1038/s41419-022-04576-4 (PMC8816910; doi:10.1038/s41419-022-04576-4)

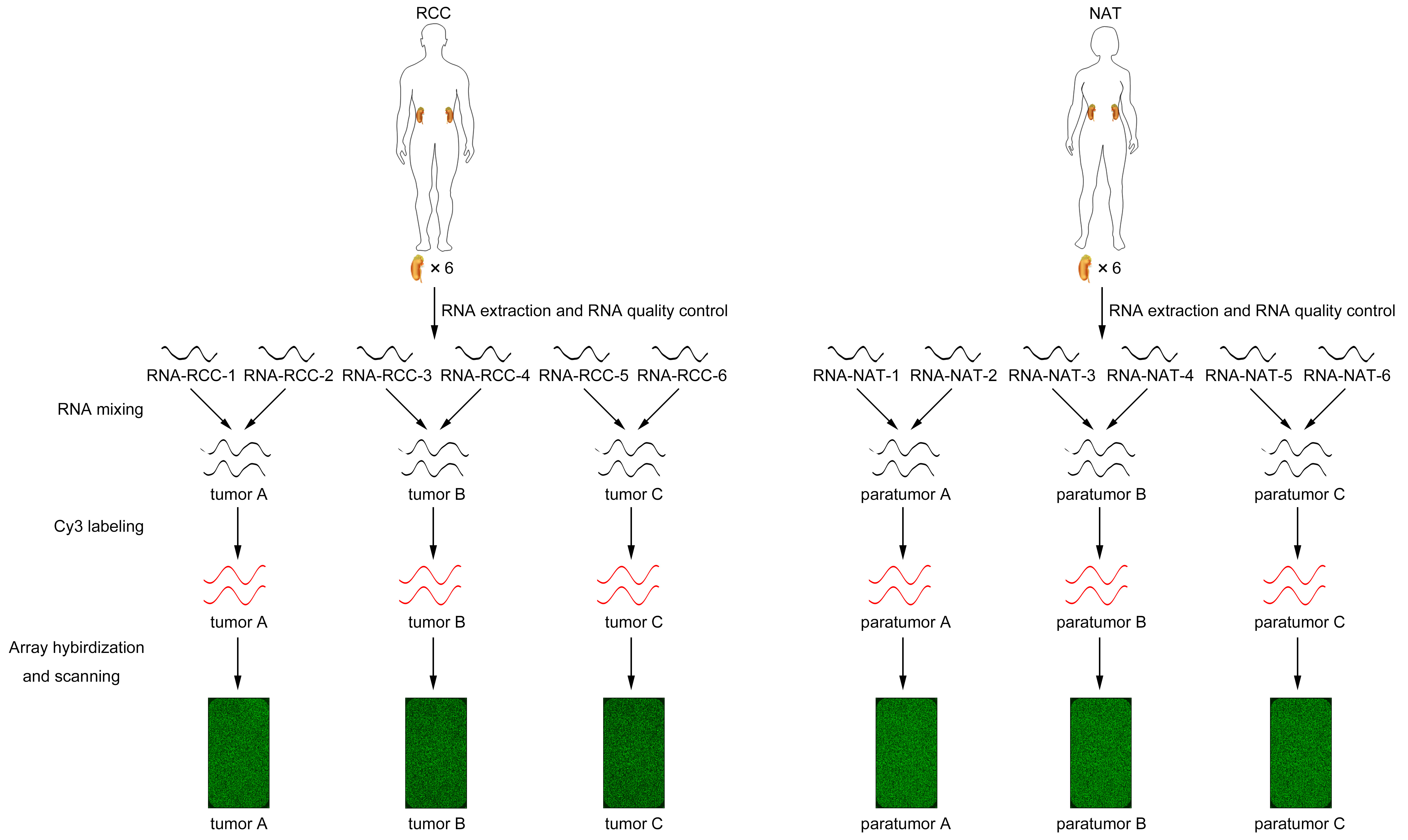

Supplement: Supplementary file 1 — Supplementary Figure 1 [file 41419_2022_4576_MOESM1_ESM.tif]

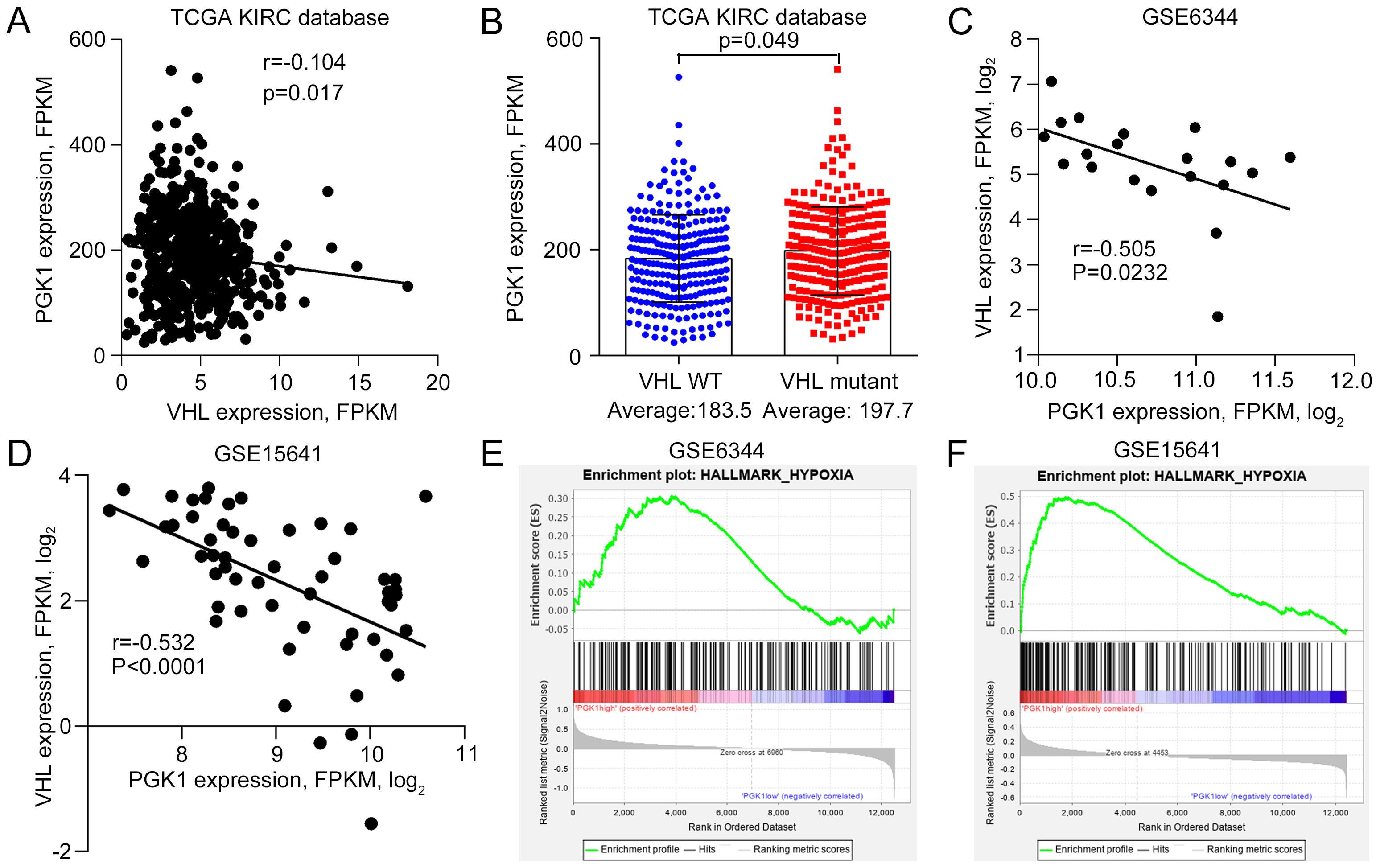

Supplement: Supplementary file 2 — Supplementary Figure 2 [file 41419_2022_4576_MOESM2_ESM.tif]
