# Supplementary material for: PGK1 contributes to tumorigenesis and sorafenib resistance of renal clear cell carcinoma via activating CXCR4/ERK signaling pathway and accelerating glycolysis
Source: Cell Death Dis. 2022 Feb 4;13(2):118. doi: 10.1038/s41419-022-04576-4 (PMC8816910; doi:10.1038/s41419-022-04576-4)
Supplement: Supplementary file 3 — Supplementary legends [file 41419_2022_4576_MOESM3_ESM.doc]

Supplementary Figure 1. Flow chart of the microarray experimental design. 6 KIRC tissues and 6 paired normal adjacent tissues were obtained for microarray analysis. Total RNA from each tissue was isolated and purified. All RNA samples were subject to quality control steps. We mixed the extracted RNA in pairs to reduce the error caused by individual differences. Labeling, array hybridization and scanning were performed following the standard protocol of Agilent Technology.

Supplementary Figure 2. PGK1 is correlated with VHL inhibition or mutation, and tumor hypoxia in KIRC tissues. (A) In TCGA-KIRC clinical samples, PGK1 expression was negatively correlated with VHL expression at the mRNA level. (B) The VHL mutation was associated with increased PGK1 expression in KIRC tumors based on the analysis of TCGA cohort of KIRC tumors, t-test. (C, D) Correlation between PGK1 and VHL mRNA expression in 10 KIRC samples and 10 normal kidney samples from GSE6344 dataset (C) and 32 KIRC samples and 23 normal kidney samples from GSE15641 dataset (D). (E) PGK1 expression was positively correlated with tumor hypoxia in KIRC through GSEA analysis of the transcriptome data in the GSE6344 dataset. (F) PGK1 expression was positively correlated with tumor hypoxia in KIRC through GSEA analysis of the transcriptome data in the GSE15641 dataset.
