# Supplementary material for: PGK1 contributes to tumorigenesis and sorafenib resistance of renal clear cell carcinoma via activating CXCR4/ERK signaling pathway and accelerating glycolysis
Source: Cell Death Dis. 2022 Feb 4;13(2):118. doi: 10.1038/s41419-022-04576-4 (PMC8816910; doi:10.1038/s41419-022-04576-4)
Supplement: Supplementary file 4 — Dataset 1 [file 41419_2022_4576_MOESM4_ESM.doc]

Supplementary Table 1. Clinical information of patients for microarray analysis

| Case no. | Gender | Age | Tumor site | Pathological classification | TNM stage |
| --- | --- | --- | --- | --- | --- |
| 1 | Male | 53 | Right kidney | ccRCC | Ⅱ |
| 2 | Male | 57 | Left kidney | ccRCC | Ⅱ |
| 3 | Male | 76 | Right kidney | ccRCC | Ⅲ |
| 4 | Male | 51 | Left kidney | ccRCC | Ⅲ |
| 5 | Female | 65 | Right kidney | ccRCC | Ⅱ |
| 6 | Female | 63 | Left kidney | ccRCC | Ⅱ |
