# Supplementary material for: PGK1 contributes to tumorigenesis and sorafenib resistance of renal clear cell carcinoma via activating CXCR4/ERK signaling pathway and accelerating glycolysis
Source: Cell Death Dis. 2022 Feb 4;13(2):118. doi: 10.1038/s41419-022-04576-4 (PMC8816910; doi:10.1038/s41419-022-04576-4)
Supplement: Supplementary file 5 — Dataset 2 [file 41419_2022_4576_MOESM5_ESM.doc]

Supplementary Table 2. RNA quality control for the microarray detection

| Sample ID | OD  260/280 ratio | OD  260/230 ratio | Concentration (ng/μL) | Volume  (μl) | Quantity (ng) | QC result  Pass or Fail |
| --- | --- | --- | --- | --- | --- | --- |
| RNA-RCC-1 | 1.93 | 2.13 | 413.12 | 80 | 33049.60 | pass |
| RNA-RCC-2 | 1.89 | 1.90 | 330.94 | 60 | 19856.40 | pass |
| RNA-RCC-3 | 1.88 | 2.15 | 418.21 | 30 | 12546.30 | pass |
| RNA-RCC-4 | 1.94 | 1.86 | 1675.45 | 80 | 134036.00 | pass |
| RNA-RCC-5 | 1.93 | 2.29 | 286.55 | 50 | 14327.50 | pass |
| RNA-RCC-6 | 1.92 | 1.02 | 182.18 | 80 | 14574.40 | pass |
| RNA-NAT-1 | 2.00 | 2.28 | 629.48 | 30 | 18884.40 | pass |
| RNA-NAT-2 | 2.00 | 2.34 | 1055.79 | 20 | 21115.80 | pass |
| RNA-NAT-3 | 1.93 | 2.36 | 600.44 | 20 | 12008.80 | pass |
| RNA-NAT-4 | 1.99 | 2.32 | 976.58 | 20 | 19531.60 | pass |
| RNA-NAT-5 | 1.99 | 2.29 | 737.79 | 30 | 22133.70 | pass |
| RNA-NAT-6 | 2.00 | 2.38 | 1079.18 | 30 | 32375.40 | pass |
